# Development of a nomogram prediction model for early identification of persistent coronary artery aneurysms in kawasaki disease

**DOI:** 10.1186/s12887-023-03876-8

**Published:** 2023-02-16

**Authors:** Xue Gong, Liting Tang, Mei Wu, Shuran Shao, Kaiyu Zhou, Yimin Hua, Chuan Wang, Yifei Li

**Affiliations:** 1 Department of Pediatrics, Ministry of Education Key Laboratory of Women and Children’s Diseases and Birth Defects, West China Second University Hospital, Sichuan University, Sichuan Chengdu, China; 2 Department of Pediatrics, West China Second University Hospital, Key Laboratory of Birth Defects and Related Diseases of Women and Children of MOE, Sichuan University, 20 3rd Section, Renmin S.Rd, Sichuan 610041 Chengdu, China

**Keywords:** Nomogram prediction, Kawasaki disease, Coronary artery aneurysms persistence, Risk factor, Decision curve analysis

## Abstract

**Background:**

Coronary artery aneurysms (CAA) persistence prediction is critical in evaluating Kawasaki disease (KD). This study established a nomogram prediction system based on potential risk factors for assessing the risk of CAA persistence in a contemporary cohort of patients with KD.

**Methods:**

This cohort comprised 105 patients with KD who had been diagnosed with CAA during the acute or subacute phase by echocardiography. The follow-up duration was at least 1 year. The clinical and laboratory parameters were compared between the CAA regression and persistence groups. Multivariable logistic regression analysis was used to identify the independent risk factors for CAA persistence, which were subsequently used to build the nomogram predictive model. Decision curve analysis was used to assess the net benefits of different nomogram scores.

**Results:**

Of these patients with CAA, 27.6% of patients presented with persistent lesions. The incidences of CAA persistence were 14.1%, 81.3%, and 100.0% in patients with small, medium, and large aneurysms, respectively. The ratio of neutrophils to lymphocytes, γ-GT, and CAA size at diagnosis were considered as the independent risk factors for CAA persistence in patients with KD. The nomogram predictive models yielded a high capability in predicting CAA persistence, based on either univariable or multivariable analyses-identified parameters, compared with using CAA size as a single predictor.

**Conclusion:**

The initial ratio of neutrophils to lymphocytes, γ-GT, and CAA size were the independent risk factors for CAA persistence in patients with KD. Nomogram scores could help elevate predictive efficacy in detecting CAA persistence.

## Background

Kawasaki disease (KD) is an acute systemic vasculitis that predominantly affects infants and children [1]. Notably, a persistent and ongoing inflammatory reaction might account for coronary artery aneurysm (CAA) development as the severe sequelae of KD. Although timely treatment with intravenous immunoglobulin (IVIG) is substantially effective, CAA still affects approximately 4.5% of patients during the acute phase of KD [2]. CAA may persist and even result in thrombosis [3–5]. Persistent CAA can be observed among 50–67% of medium-sized CAA, and 14–20% of them would progress into arterial stenosis due to the presentation of thrombus after the disorder of coronary morphology in patients with CAA after the acute phase of KD [6]. The long-term prognosis of CAA predominately relies on the consequences of CAA persistence and progression in KD, including morbidity, cardiac events, and even death [7–13]. Moreover, the American Heart Association (AHA) scientific statement on cardiovascular risk reduction in high-risk pediatric patients [14] and the National Heart, Lung, and Blood Institute–commissioned Expert Panel for Cardiovascular Health and Risk Reduction in Children and Adolescents [15] emphasize that KD with concomitant persistent CAA was classified as a high-risk condition.

Therefore, early identification and appropriate interventions of persistent CAA are crucial and could improve the prognosis of patients with KD [11, 16]. In clinical practice, a useful tool for predicting persistent CAA in KD is urgently needed, as patients may benefit from personalized management according to corresponding risk stratification. Several studies have identified the risk factors for CAA persistence and proposed several risk-scoring systems [17–20]. Encouragingly, a nomogram prediction score is widely identified as a useful model for predicting the prognosis of different diseases in clinical practice [21]. Nomogram prediction models have been demonstrated to perform much better in assessing the prognosis of several conditions than other models [22, 23]. Accordingly, a nomogram model may be a potential tool for predicting persistent CAA in KD. Herein, this study aimed to identify the risk factors of persistent CAA and attempted to establish a nomogram prediction model to evaluate the risk of persistent CAA in KD. Additionally, this nomogram model may provide advanced information for clinical management of persistent CAA in KD.

## Methods

### Questionnaire and purposes

Due to the unsatisfactory efficiency of current predictive models of CAA lesions in KD, this study sought to establish a reliable nomogram prediction system to assess the possibilities of CAA persistence associated with KD. The study was a prospectively designed observational study, and all participants were enrolled from January 2016 to December 2020.

### Patient population

This prospectively designed, observational, single-center study included all patients with KD who visited West China Second University Hospital, Sichuan University from January 2016 to December 2020. Participants were followed up for at least 1 year; and during the follow-up, they underwent at least three echocardiographic assessments. This study was conducted under strict adherence to the STROBE statement. The Ethics Committee of the West China Second Hospital of Sichuan University approved this study (approval number 2014-034). Informed written consent was obtained from the parents after the nature of this study had been fully explained to them. All questionnaires were pretested and revised accordingly. Two pre-trained physicians collected data and followed up on participants. Any related questionnaires were double-checked to assure accuracy. All clinical data were confirmed by electronic medical records and the study’s follow-up database.

### Inclusion and exclusion criteria

To recruit candidates for further analysis, we used the following inclusion criteria: (1) Confirmed diagnosis of KD by two physicians using the diagnostic criteria for complete or incomplete diagnostic standards recommended by the Japanese Circulation Society (2013) for diagnosis, treatment, and long-term management of KD; (2) identification of CAA either by transthoracic echocardiography or transcatheter angiography and identification of CAA onset within the acute or subacute phase of KD; (3) well-collected and completed programmed questionnaires, basic essential information, clinical manifestation, hematological examination results, therapeutic procedure, echocardiography results, and follow-up outcomes; (4) completed baseline hematological examinations during acute phase of KD before IVIG administration; (5) age of 2–6 years, which is the age range with the highest incidence of KD, to balance bias from high risk ages; and (6) absence of CAA lesions from previous episodes of KD, if patient had previous more than one episode of KD. The exclusion criteria included the following: (1) presence of cardiovascular malformations; (2) history of an autoimmune disease prior to KD onset; (3) treatment with anticoagulant or antiplatelet medication prior to KD onset; (4) history of cardiac surgeries; (5) suspicion of myocarditis prior to KD onset; (6) use of glucocorticoids prior to IVIG administration; (7) use of monoclonal antibodies, including tumor necrosis factor (TNF)-α and interleukin (IL)-6 antibodies; (8) diagnosis of macrophage activation syndrome or hemophagocytic lymph histiocytosis secondary to KD; (9) new onset of coronary artery lesions after persistent CAA; and (10) absence of echocardiographic records for ≥ 1 year.

### Therapeutic and follow-up procedure

All patients with KD were treated with high-dose IVIG (2 g/kg given as a single intravenous infusion) and 30–50 mg/kg/day high dose aspirin. Those with recrudescent or persistent fever ≥ 36 h after the first dose of IVIG infusion were treated with a second dose of 2 g/kg IVIG. Methylprednisolone (30 mg/kg/day for three consecutive days) followed by oral prednisone tapered over 7 days would be considered after the second IVIG administration. The first day of fever was defined as day 1 of disease onset, and IVIG resistance was defined as persistent or recurrent fever (temperature ≥ 38.0 °C orally) or presence of other clinical signs of KD for at least 36 h but not longer than 7 days after initial IVIG administration. The patients were discharged from the hospital after temperature remained normal for > 48 h and hematological examination findings returned to normal. Follow-up began from day of hospital discharge. All participants underwent echocardiographic evaluations after 2 weeks, 1 month, 2 months, 3 months, 6 months, and 12 months; findings from these evaluations were documented from the end of the subacute phase.

### Echocardiographic evaluation

Two well-trained pediatric physicians performed all echocardiographic evaluations. The physicians involved in the evaluation were blinded to the clinical presentations of participants. The first echocardiographic evaluation was performed before IVIG administration, and the second evaluation was performed during the subacute phase or before hospital discharge. We defined the morphology and luminal dilation of the coronary artery, which were documented as aneurysm size, as the most severe condition between the acute and subacute phases of KD. At least two echocardiographic evaluations were performed to match the minimal requirement for first-month follow-up. Additionally, the first observation of pre-existing CAA recovery to a normal size was considered as the time for regression. If CAA persisted for 12 months, it was defined as a persistent lesion. Based on the Japanese Circulation Society criteria [24], CAA was defined as a coronary artery branch internal luminal diameter of > 3 mm in a child under the age of 5 years, an arterial diameter of > 4 mm in a child ≥ 5 years of age, or when an arterial segment was 1.5 times its adjacent segment. The CAAs were classified as small aneurysms (localized dilatation with ≤ 4 mm internal diameter), medium aneurysms (aneurysms with an internal diameter from > 4 mm to < 8 mm), and giant aneurysms (aneurysms with an internal diameter of ≥ 8 mm).

### Risk factor analysis

All parameters involved were used to predict the risk of CAA persistence after 1 year of follow-up. First, the basic clinical characteristics and hematological examination results of participants were recorded (Table 1). Thereafter, univariable analysis was performed between patients with CAA persistence and patients with regression. Multivariable analysis was completed using logistic regression to identify the independent factors among the significant results obtained from the univariable analysis. Nomograms were constructed based on findings from the univariable and multivariable analyses. The final prognostic nomogram model was selected using a backward step-down selection process and the Akaike information criterion. Thereafter, receiver operating characteristic curves (ROC) were used to identify the predictive value of the risk factors and nomogram scores that were found by comparison. Decision curve analysis (DCA) was performed to evaluate the efficacy of nomogram prediction. Finally, calibration plot was used to test the accuracy of Multi_nomo and Uni_nomo scores.

**Table 1:** Univariable analysis for risk factors of coronary artery aneurysm persistence

Variables	CAA regression (*n* = 76)	CAA persistence (*n* = 29)	Sig.
Gender			0.633
Female	23 (30.27%)	7 (24.14%)	
Male	53 (69.73%)	22 (75.86%)	
Complications in CNS	21 (27.63%)	7 (24.14%)	0.808
Incomplete KD	40 (52.63%)	16 (55.17%)	0.831
IVIG resistance	19 (25.00%)	9 (31.03%)	0.623
Relapsed KD	29 (38.16%)	8 (27.58%)	0.366
White blood cell (×10^9^/L)	14.25 ± 5.07	14.07 ± 5.98	0.882
Neutrophils (%)	64.31 ± 13.96	74.42 ± 11.01	0.001*
Lymphocyte (%)	25.37 ± 11.92	18.32 ± 8.71	0.004*
Ratio of neutrophils to lymphocyte	3.65 ± 3.76	5.73 ± 4.19	0.016*
Monocyte (%)	10.31 ± 4.08	7.25 ± 3.97	0.001*
Hemoglobin (g/L)	107.61 ± 12.06	111.17 ± 10.85	0.167
Platelet (×10^9^/L)	352.14 ± 150.97	394.34 ± 166.93	0.216
Ratio of platelet to lymphocyte	125.33 ± 82.08	186.91 ± 129.49	0.005*
CRP (mg/L)	87.35 ± 55.21	78.68 ± 48.41	0.459
ESR (mm/h)	59.97 ± 28.62	70.84 ± 32.34	0.115
Alanine aminotransferase (ALT, U/I)	57.92 ± 65.26	71.31 ± 117.60	0.461
Aspartate aminotransferase (AST, U/I)	50.29 ± 59.88	104.03 ± 130.87	0.005*
Ratio of AST to ALT	1.28 ± 0.77	2.41 ± 1.82	0.000*
Total Bilirubin (umol/L)	9.37 ± 10.06	8.53 ± 14.14	0.741
Direct bilirubin (DBIL, umol/L)	5.94 ± 8.24	4.74 ± 12.30	0.598
Indirect bilirubin (umol/L)	4.21 ± 3.47	3.87 ± 3.64	0.682
Albumin (ALB, g/L)	35.72 ± 6.52	36.99 ± 5.94	0.366
Prealbumin (PA, g/L)	62.83 ± 31.37	72.69 ± 46.85	0.422
γ-Glutamyl transpeptidase (γ-GT, U/L)	88.26 ± 92.75	41.76 ± 31.24	0.009*
Lactate dehydrogenase (LDH, U/L)	383.79 ± 193.87	394.17 ± 202.01	0.812
Urea nitrogen (UN, mmol/L)	3.26 ± 1.86	3.45 ± 3.11	0.723
Creatinine (Cr, umol/L)	28.48 ± 11.55	31.17 ± 14.99	0.344
Serum cystain C (CysC, umol/L)	1.01 ± 0.38	0.94 ± 0.52	0.667
K+ (mmol/L)	4.26 ± 0.58	4.18 ± 0.68	0.568
Na+ (mmol/L)	134.86 ± 15.03	137.31 ± 4.73	0.393
P- (mmol/L)	1.33 ± 0.28	1.41 ± 0.27	0.201
Cl- (mmol/L)	102.39 ± 3.83	102.22 ± 3.84	0.844
Ca2+ (mmol/L)	2.28 ± 0.15	2.28 ± 0.18	0.826
Mg2+ (mmol/L)	0.93 ± 0.14	0.92 ± 0.09	0.709
Total cholesterol (TC, mmol/L)	3.04 ± 0.82	3.40 ± 0.69	0.184
Triglyceride (TG, mmol/L)	1.38 ± 0.50	1.72 ± 1.46	0.277
HDL-C (mmol/L)	0.51 ± 0.18	0.63 ± 0.29	0.072
LDL-C (mmol/L)	2.23 ± 0.81	2.24 ± 0.87	0.982
PT (s)	14.06 ± 2.71	13.15 ± 1.18	0.140
APTT (s)	36.60 ± 8.87	35.57 ± 6.68	0.638
Fibrinogen (Fg, mg/dL)	531.19 ± 147.43	556.95 ± 133.44	0.497
D-dimer (mg/L)	2.01 ± 1.44	1.86 ± 1.76	0.789
CTnI (ug/L)	0.02 ± 0.04	0.02 ± 0.02	0.740
NT-BNP (pg/ml)	2384.06 ± 4601.73	2279.28 ± 5989.78	0.941
Cardiac enlargement	13 (17.11%)	3 (10.34%)	0.547
Valver regurgitation	16 (21.05%)	4 (13.79%)	0.289
Number of lesions			0.000*
1	67 (88.16%)	15 (51.72%)	
2	6 (7.89%)	8 (27.60%)	
3	3 (3.95%)	3 (10.34%)	
4	0 (0%)	3 (10.34%)	
Aneurysm classification			0.000*
Small	73 (96.05%)	12 (41.38%)	
Medium	3 (3.95%)	13 (44.83%)	
Large	0 (0%)	4 (13.79%)	
Location of aneurysm			0.000*
Left coronary artery (LCA)	57 (75.00%)	11 (37.93%)	
Left anterior descending artery (LAD)	5 (6.58%)	5 (17.24%)	
Left circumflex artery (LCX)	0 (0.00%)	0 (0.00%)	
Right coronary artery (RCA)	14 (18.42%)	13 (44.83%)	
Aneurysm size	3.37 ± 0.34	5.10 ± 2.52	0.000*

### Statistical analysis

All data analyses were performed using SPSS 22.0 (SPSS Inc. Chicago, Illinois, United States of America). Continuous variables are presented as mean ± standard deviation, whereas categorical variables are expressed as frequencies. Differences between two groups were assessed using independent t-tests or Mann-Whitney U tests for continuous variables and chi-squared test or Fisher’s exact test for categorical variables. ROC analysis was used to determine the predictive validity of candidate risk factors for persistent CAA. Statistical significance was defined by *p*-values < 0.05. Nomograms and DCA were established and performed, respectively, using the *rms* and *rmda* packages of R version 3.3.2 (http://www.r-project.org).

## Results

### Study population

**Fig. 1:**
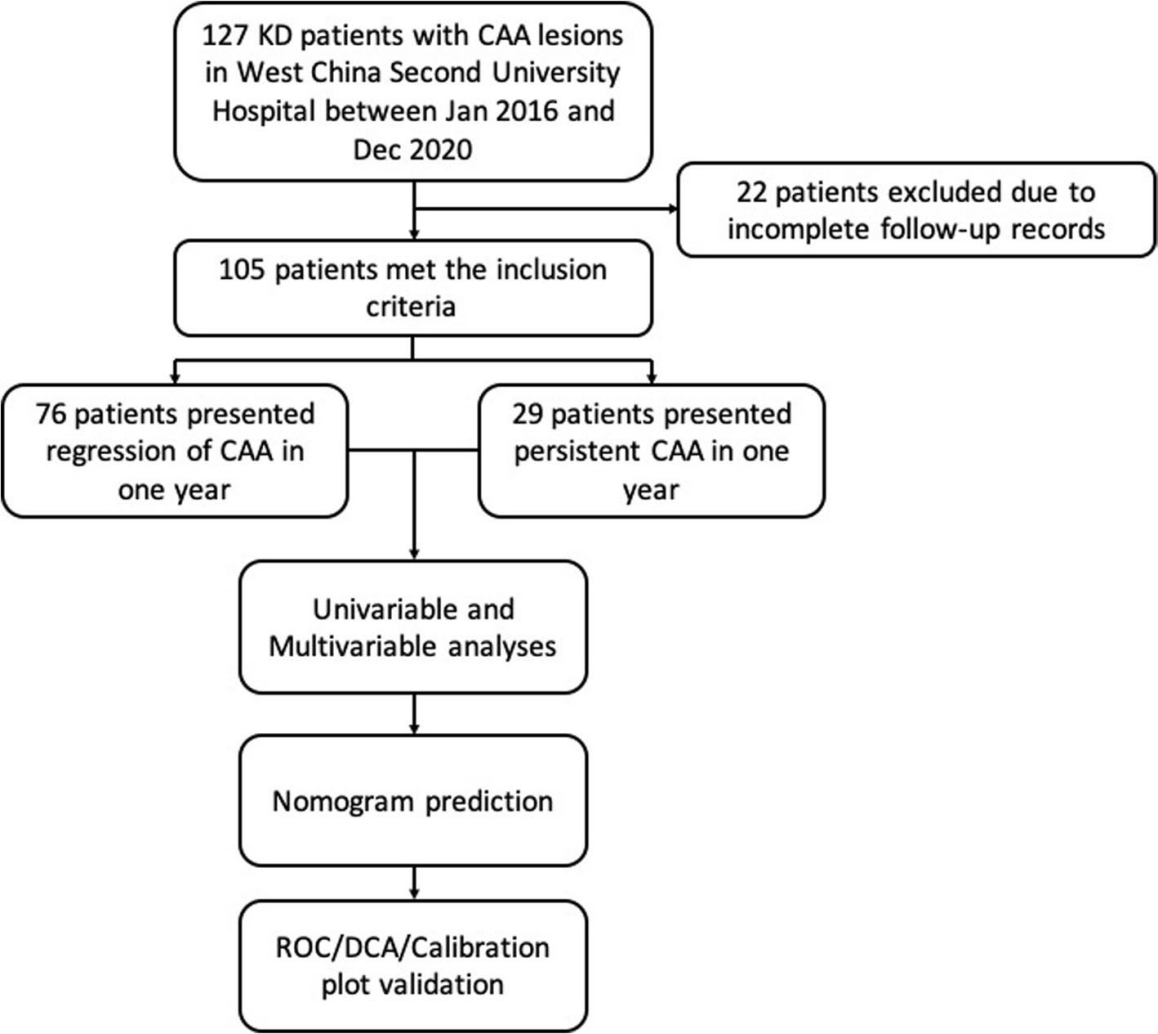
The flow chart of this study. KD, kawasaki disease; CAA, Coronary artery aneurysms; ROC, receiver operating characteristic curves; DCA, decision curve analysis

A total of 127 patients with observed CAA between acute and subacute phases were included in this study. However, 105 patients were finally selected for further analysis based on the inclusion and exclusion criteria. Twenty-eight patients suffered central neurological system complications. Fifty-six patients were diagnosed with incomplete KD. Additionally, 28 patients demonstrated IVIG resistance, and 37 relapse cases were recorded. Twenty-nine patients had persistent CAA, whereas 76 patients presented with CAA regression. Follow-up time was 12–58 months. Only three patients underwent transcatheter angiography, which demonstrated a similar coronary artery morphology as that revealed by echocardiography. No mortalities were recorded during follow-up. The flow chart of cohort establishment and analysis is illustrated in Fig. 1.

### Risk factors related to persistent CAA

CAA-related factors were analyzed according to the processes described in flow charts 1 (univariable analysis) and 2 (multivariable analysis). As shown in Table 1, univariable analysis revealed several variables associated with persistent CAA, including percentage of neutrophils (*p* = 0.001), percentage of lymphocytes (*p* = 0.004), ratio of neutrophils to lymphocytes (*p* = 0.016), percentage of monocytes (*p* = 0.001), ratio of platelets to lymphocytes (*p* = 0.005), AST (*p* = 0.005), ratio of AST to ALT (*p* = 0.000), γ-GT level (*p* = 0.009), number of CAA lesions (*p* = 0.000), classification of aneurysm (*p* = 0.000), location of aneurysm (*p* = 0.000), and size of aneurysm size (*p* = 0.000). Thereafter, these variables were further analyzed using multivariable analysis. However, only three variables were identified as independent factors for persistent CAA (Table 2). They included ratio of neutrophils to lymphocytes (OR = 1.243, *p* = 0.008, 95%CI = 1.058–1.460), γ-GT level (OR = 0.977, *p* = 0.027, 95%CI = 0.957–0.997), and aneurysm size (OR = 341.173, *p* = 0.000, 95%CI = 20.403–705.038).

**Table 2:** Univariable analysis for risk factors of coronary artery aneurysm persistence

Variables	OR	Sig.	Lower 95% CI	Upper 95%CI
Neutrophils (%)	1.126	0.624	0.701	1.809
Lymphocyte (%)	0.974	0.918	0.591	1.606
Ratio of neutrophils to lymphocyte	1.243	0.008*	1.058	1.460
Monocyte (%)	1.015	0.955	0.599	1.720
Ratio of platelet to lymphocyte	1.009	0.516	0.983	1.035
AST	1.018	0.134	0.995	1.041
Ratio of AST to ALT	5.007	0.070	0.874	28.681
γ-GT	0.977	0.027*	0.957	0.997
Number of lesions	0.818	0.891	0.047	14.371
Aneurysm classification	2.746	0.111	0.793	9.503
Location of aneurysm	1.862	0.835	0.005	647.060
Aneurysm size	341.173	0.000*	20.403	705.038

*ALT * Alanine aminotransferase, *AST *Aspartate aminotransferase, *γGT* γ-Glutamyl transpeptidase

### Nomogram calculation

**Fig. 2:**
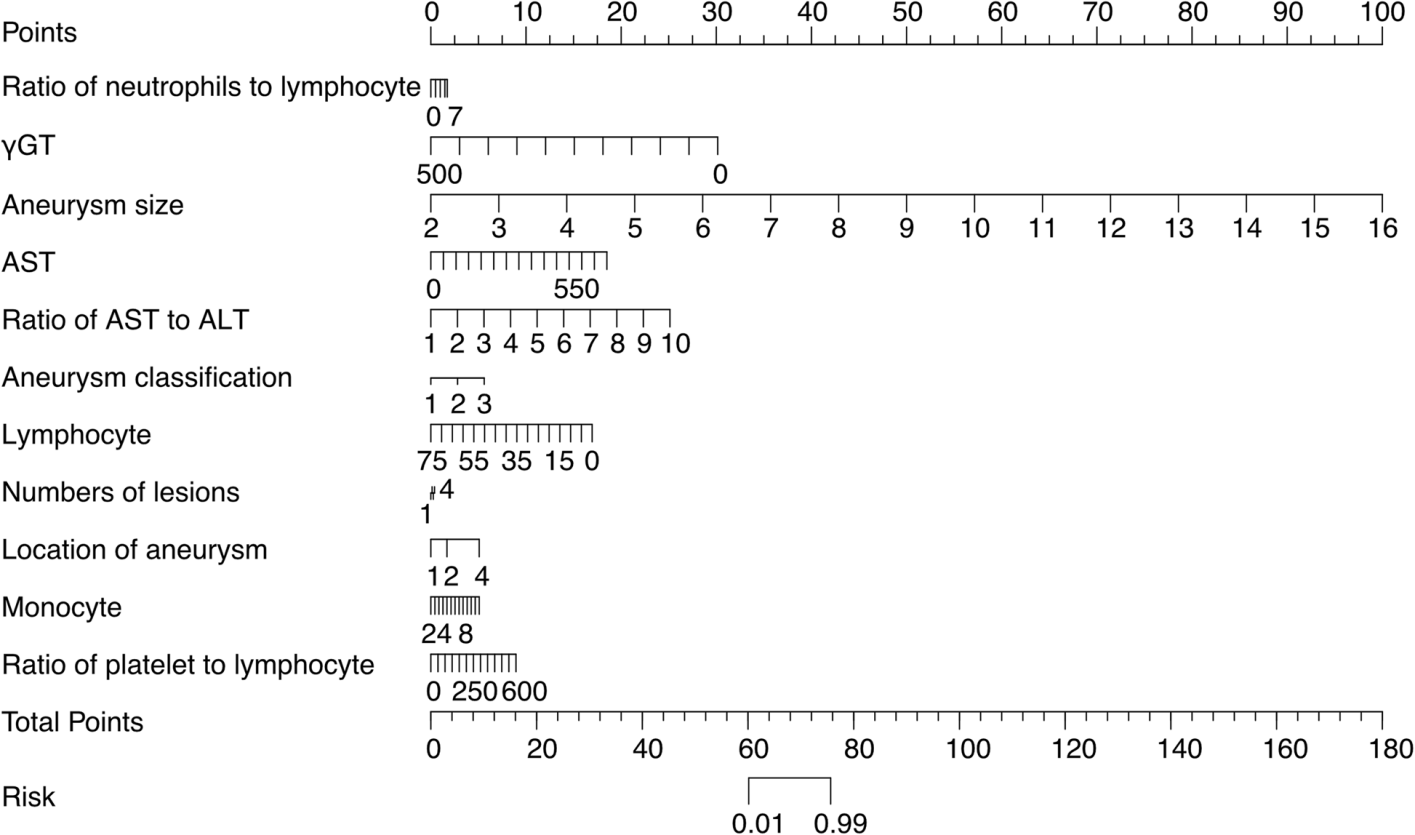
The nomogram prediction scores of univariable analysis

**Fig. 3:**
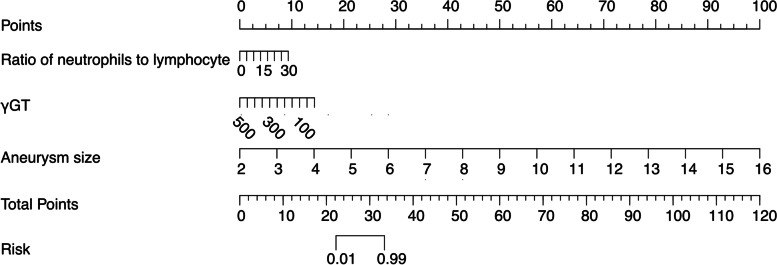
The nomogram prediction scores of multivariable analysis

Nomogram predictions were made based on findings from the univariable and multivariable analyses. The nomogram formula, which was based on the 11 variables identified by univariable analysis and three independent risk factors identified by multivariable analysis, is presented in Figs. 2 and 3. Using the established nomogram formula, we calculated the specific nomogram score of univariable factors (Uni_nomo) for every enrolled individual, and the average Uni_nomo scores were 44.85 ± 18.28 among patients with CAA persistence and 27.66 ± 5.87 among patients with CAA regression. For the nomogram scores based on multivariable factors (Multi_nomo), the average Multi_nomo scores were 44.24 ± 18.37 among patients with CAA persistence and 28.17 ± 4.53 among patients with CAA regression.

### ROC and DCA evaluations

**Fig. 4:**
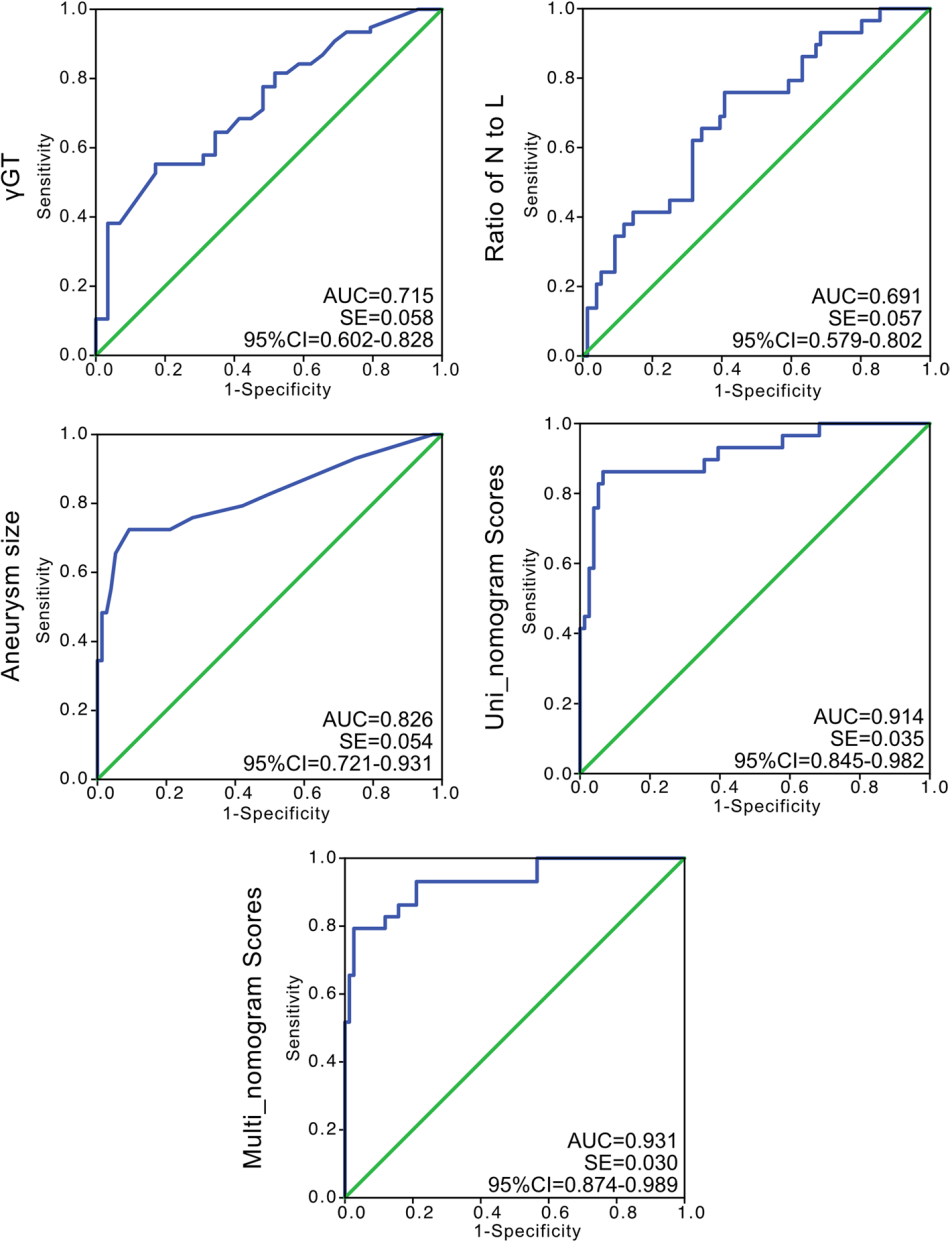
The ROC curves of γ-GT, ratio of neutrophil to lymphocyte, CAA size, Uni_nomogram scores and Multi_nomogram scores

**Fig. 5:**
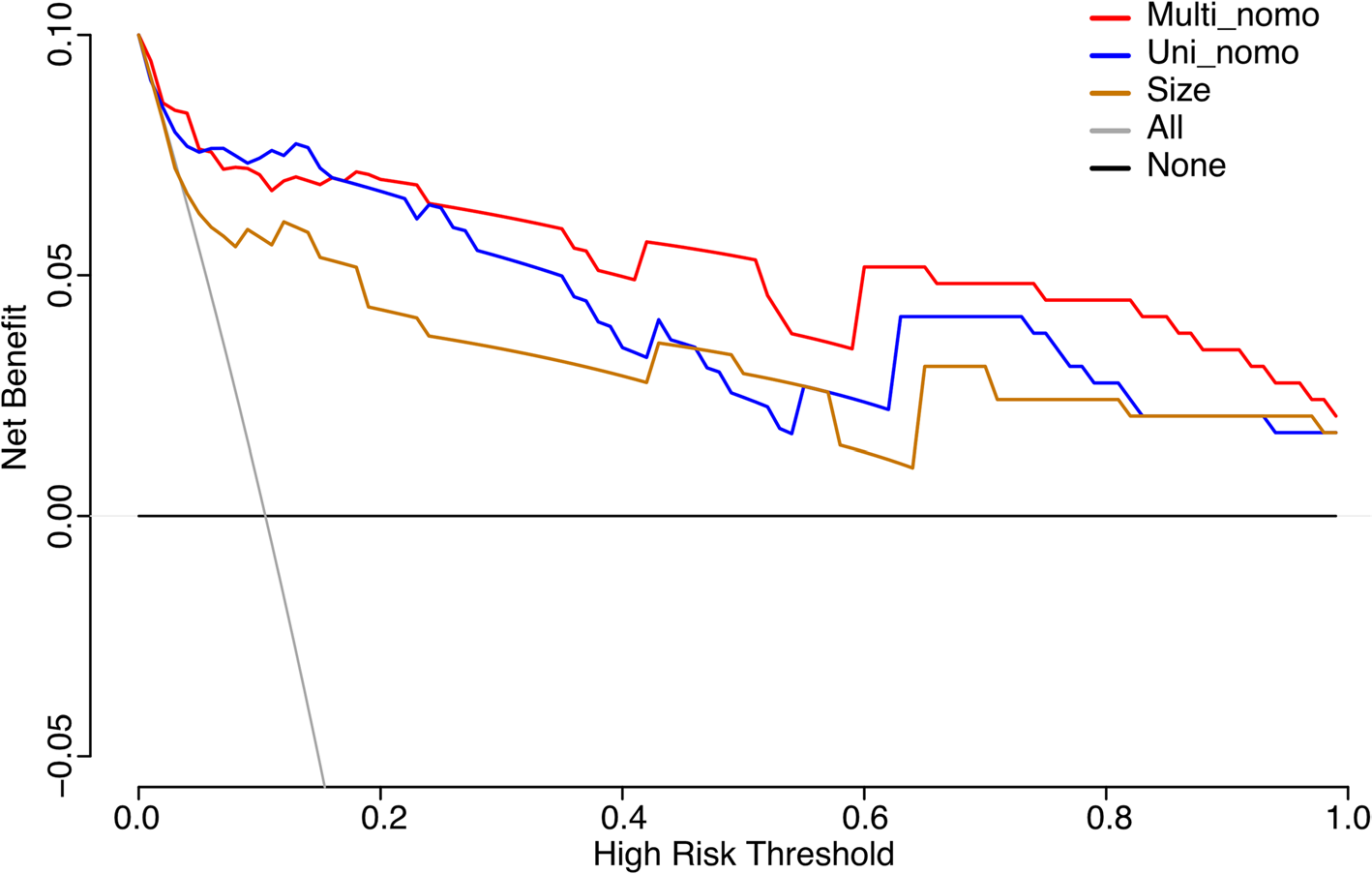
Decision curve analysis based on CAA size, Uni_nomogram scores and Multi_nomogram scores at 10% CAA incidence. The curves “none” or “all” were presented as referencing lines, which indicated no one or all patients would benefit from clinical decision based on current strategy at any high risk threshold

**Fig. 6:**
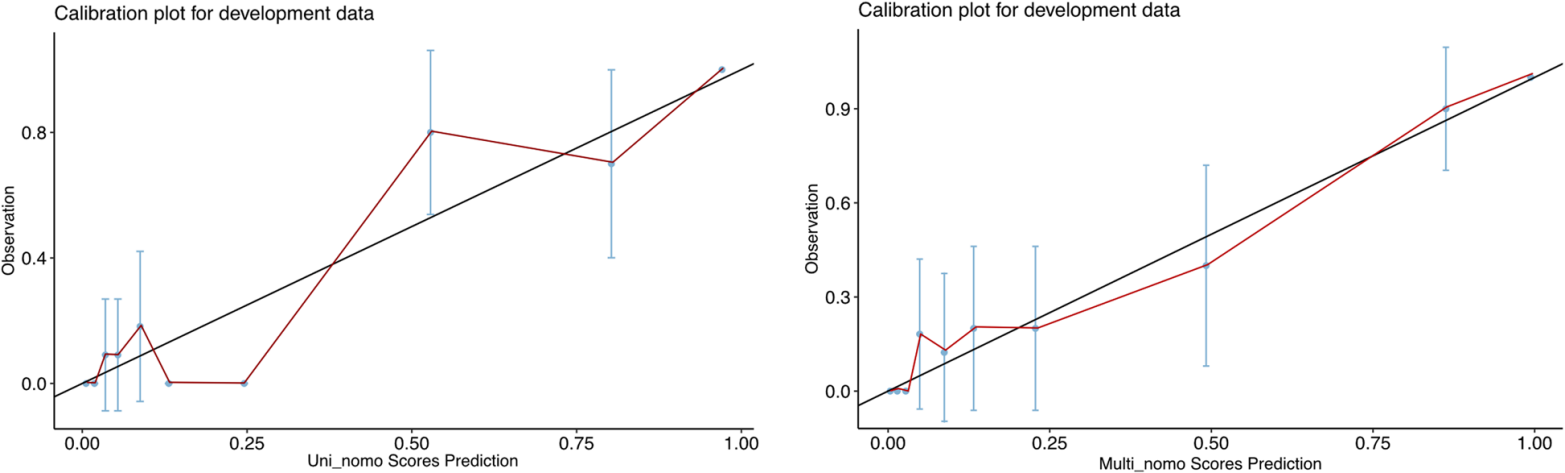
Calibration plot analyses for Uni_nomogram and Multi_nomogram scores

ROC curves were used to validate the efficacy of independent factors and nomogram scores in predicting persistent CAA (Fig. 4). However, both γ-GT level (AUC = 0.715, SE = 0.058, 95% CI = 0.602–0.828) and ratio of neutrophils to lymphocytes (AUC = 0.691, SE = 0.057, 95% CI = 0.579–0.802) demonstrated an intermittent efficacy in distinguishing persistent CAA at early term. Using aneurysm size only, the predictive efficacy was improved (AUC = 0.826, SE = 0.054, 95% CI = 0.721–0.931). Furthermore, the ROC curves of Uni_nomo scores (AUC = 0.914, SE = 0.035, 95% CI = 0.845–0.982) and Multi_nomo scores (AUC = 0.931, SE = 0.030, 95% CI = 0.874–0.989) presented an excellent efficacy in detecting persistent CAA. Lastly, DCA was used to assess the advantages of the nomogram scores. The DCA was performed using the estimated CAA incidence of 10% among all patients with KD. The curves “none” or “all” were presented as referencing lines, which indicated that no one or all patients would benefit from clinical decisions based on current strategy at any high-risk threshold. Additionally, the Multi_nomo scores revealed an advantage in distinguishing persistent CAA among most of the range of the high-risk threshold, which demonstrated the advantage in the application of Multi_nomo scores (Fig. 5). The calibration plot analyses demonstrated a good accuracy of Multi_nomo score, whereas the Uni_nomo score failed to present a satisfied accuracy (Fig. 6).

## Discussion

Clinically, persistent CAA prediction is necessary for KD management. To the best of our knowledge, this study was the first to establish a nomogram model for persistent CAA prediction in patients with KD. In this cohort, we identified γ-GT, ratio of neutrophils to lymphocytes, and aneurysm size as the independent risk factors for persistent CAA by multivariable analysis. Aneurysm size dominantly contributed to the persistence of CAA. However, ratio of neutrophils to lymphocytes and γ-GT values could help predict the possibility of CAA persistence, as the ROC curve of nomogram scores covered more area than that of aneurysm size only. The elevated ratio of neutrophils to lymphocytes and γ-GT level were associated with advanced inflammation attacks, which contributed to CAA persistence. Aneurysm size, as a single variable, was more reliable in predicting persistent CAA than γ-GT level or ratio of neutrophils to lymphocytes. However, a single variable is not efficient enough to achieve high sensitivity and specificity. Therefore, the nomogram prediction model would contribute to assessing the possibility of persistent CAA based on a calculated formula with multiple variables.

Accordingly, we validated the efficacy of Uni_nomo scores (based on 11 univariable analysis-identified variables) and Multi_nomo scores (based on three independent risk factors identified by multivariable analysis) in persistent CAA prediction. Besides, we used the selected variables from univariable and multivariable analyses to establish nomograms. However, the Uni_nomo and Multi_nomo scores covered similar AUCs under ROC. Therefore, the three independent variables identified by multivariable analysis were more efficient for predicting CAA persistence and more applicable than Uni_nomo, which comprised 11 variables.

Three pathological processes underlie the occurrence of coronary artery lesions in KD. As the most severe consequence of KD, CAA has been considered as the major cause for morbidity and mortality among patients with KD [25], who are mainly affected by initial inflammation. Therefore, enormous clinical effort has been made to identify any potential risk factors for CAA formation, including sex [26], strategy of IVIG treatment [26], IVIG resistance [27], duration of fever [27], extreme age (< 1 year or > 9 years) [28, 29], tachycardia [30], and any changes in blood test results of increased C-reactive protein (CRP) [26, 31], n-terminal pro-brain natriuretic peptide [32], neutrophil-to-lymphocyte ratio [31], hypoalbuminemia, total bilirubin, and platelet count. Several other evaluation score systems for CAA prediction have been developed. However, no standard system for predicting persistent CAA has been established [26].

A previous study demonstrated that neutrophil-to-lymphocyte ratio > 1 at two days post-IVIG treatment was an independent risk factor for CAA [33]. It may be associated with the subsequently persistent inflammation that occurs after IVIG administration, along with the initial inflammation reaction [19]. Moreover, the role of neutrophils is critical in acute inflammation and suggests extensively severe acute inflammation, which might account for CAA development. The aforementioned study confirmed that the neutrophil-to-lymphocyte ratio represents the initial status of inflammation activity in patients with KD. Besides, the alternation of γ-GT was not only related with disorders of the biliary system [34]. The expression of γ-GT level was associated with sialylation of gangliosides, which inhibited IVIG-mediated neutrophil apoptosis and elevated related inflammation activity [35]. Taken together, CAA size, recorded at the end of the acute phase of KD, was identified as a dominant factor in predicting CAA persistence. However, the ROC curve of CAA size in predicting persistence only covered 82.6% of AUC, showing a great potential for improving the efficiency of the prediction system. Therefore, the nomogram prediction score, as an alternative for determining CAA persistence using a calculated formula with multiple factors, increases the accuracy of the prediction system.

To date, initial inflammatory activity and hemodynamic status are suspected to be partly responsible for CAA persistence and CAA progression. An electron microscopy study demonstrated that subacute/chronic vasculitis could persist for months to years and was responsible for progressive arterial stenosis and thrombosis development [36]. Therefore, the established nomogram prediction systems used a series of variables indicating coronary artery morphology, inflammation activity, and endothelial cells injuries. Nomogram prediction scores based on univariable and multivariable analyses demonstrated a high efficiency in assessing the middle term of clinical outcomes. Such results elucidated the advantages of nomogram prediction systems, which incorporated multiple variables in determining CAA persistence, over any single independent risk factor. Moreover, DCA based on the nomogram prediction system showed that the combination of CAA size and laboratory parameters (neutrophil-to-lymphocyte ratio and γ-GT) has a higher net benefit in predicting CAA persistence than CAA size, as a single parameter. Therefore, this nomogram model is a potential powerful tool for predicting CAA persistence and may provide some information for clinical management. Although CAA size contributed dominantly to the predictive efficacy of nomogram scores, the other parameters could partially improve the predictive power in persistent CAA. Moreover, more variables should be included to build a more efficient nomogram scoring system. Therefore, CAA size could help predict CAA persistence in a simple manner with acceptable efficacy. However, a nomogram formula could provide more accurate detailed predictions in detail, if more parameters are included.

This study had some potential limitations. First, selection bias might have occurred because this study was conducted in a single institution. Besides, we comprehensively and systematically analyzed the risk factors for CAA persistence using the nomogram model for the first time by incorporating both clinical and laboratory parameters in the models. Second, the follow-up time in this cohort was not long enough, compared to previous studies focusing on the process of CAA. A large sample-size prospective cohort is required to validate the efficacy of the proposed nomogram prediction in future studies. Third, our study commenced in 2016, way before Z scores were used for CAA evaluation in recent times. Fourth, data on height and weight were obtained from medical records but not verified by a specialist, which could be a source of bias in calculating Z scores. Therefore, in this study, we only included verified parameters for further analysis and used absolute value to define CAA.

## Conclusion

Neutrophil-to-lymphocyte ratio, γ-GT level, and CAA size at the end of the acute phase were the independent risk factors for CAA persistence in patients with KD. A nomogram prediction model was established successfully. Both nomogram systems based on univariable and multivariable analyses were more efficient in predicting persistent CAA than the application of CAA size only. However, the evaluation of CAA size still made a major contribution in the nomogram prediction formula.

## Data Availability

All the data had been presented in the manuscript. Other data sets used in this study are available from the corresponding author upon reasonable request.
